# Collectanea: Pathology and Practice

**Published:** 1834-04-01

**Authors:** 


### COLLECTANEA.

### PATHOLOGY AND PRACTICE.

### ON THE APPLICATION OF THE TOURNIQUET IN PARALYSIS.

WE have been favoured with a communication by Mr. Valentine, of Somerton, who informs us that he has employed the tourniquet with success in a case of paralysis. He was induced to do so, by an account of its beneficial effects in the hands of Dr. Calhoun, of Philadelphia, which he found in the Medico-Chirurgical Review. We subjoin Mr. Valentine's case, which is certainly a very interesting one.

Mary Dampier, æt. forty, far advanced in her first pregnancy, fell down in a fit of apoplexy, on the 19th of November last. Sense and motion were lost for two hours, when she recovered in a great degree, but had still some remaining vertigo and inclination to sleep, the mouth being also drawn to one side, and the left arm paralysed. She was bled, leeched, blistered, and mercurialized; under which treatment all the symptoms were removed, except the paralysis of the arm and hand; for which she was directed to use friction with the linim. volat., which produced scarcely any benefit.

Having met with some observations on the use of the tourniquet in paralytic affections, I resolved on giving it a trial. All medicines and applications being discontinued, it was first applied on the 9th of December, as high in the arm as possible, for about twenty minutes: the effect it produced was surprising, the power of raising the limb being immediately increased on removing the tourniquet. The same plan was adopted daily till January 1, sometimes twice and three times a day, each application lasting from fifteen to twenty minutes. The motion of the whole extremity by that time was fully restored. The only immediate effects of the tourniquet were an increase of power and a greater sensation of heat. No alteration in the pulse was detected either before or after each application of the instrument. The poor woman was safely delivered on the 2d of January, and is able to dress and take care of her child.

### CASE IN WHICH THE OS UTERI WAS NEARLY OBLITERATED.

Mr. Wm. Taylor, of Stamford street, has obligingly favoured us with the details of a very remarkable case which lately occurred in his practice.

Mrs. ——, æt. thirty-five, was taken in labour, Feburary 1st, 1834. She had been married nine years, and had never borne a living child, but was said to have miscarried once, though the facts to be subsequently narrated seem to render this improbable. Mr. Taylor made an examination, but no os uteri was to be felt. The rigidity of the external parts, and other reasons, induced him to abstract twenty ounces of blood, and he also gave the patient an ounce of castor oil. The bowels were opened, and the head of the child continued to descend; but, as matters were still in a very unsatisfactory state, Mr. Taylor deemed it prudent to call Dr. Blundell into consultation. This eminent accoucheur being likewise unable to discover any os uteri, it was determined to make an incision into the uterus.

The patient being brought to the foot of the bed, with the legs drawn up towards the abdomen, an exceedingly small opening into the uterus was discovered, just large enough to admit the point of the finest probe. "A longitudinal incision was made from the symphysis to the rectum, and then a crucial incision, as extensive as the parts would admit." No hemorrhage followed. The pains naturally slackened from the alarm of the patient, and the ergot of rye was administered in large doses. The pains were increased in force, but were still incapable of expelling the fœtus, so that Mr. Taylor thought it necessary to open the head. The patient passed a good night, and every thing went on well till the fourth day, when she was attacked with peritonitis. It was easily subdued, however, and her recovery was perfect. The uterine discharge ceased at the end of three weeks. The catamenia have since appeared, and their flow has been unattended with pain, though Mrs. —— had previously suffered from dysmenorrhœa in its acutest form.

### CASE OF HYPOCHONDRIASIS.

I knew a gentleman who desired a medical man to send him some medicine to take, "for he had seven devils." The doctor said he did not like to take any man's word upon such a subject, and wished to investigate the case personally. He called on the patient, and having examined him, said, "Seven devils! you have eight, and the eighth is worse than all the others put together." It was agreed between them that the doctor should adopt some means of driving out these devils; and the first was accordingly driven out by a slight electric shock, and the others in succession by shocks increased in power, till only the eighth devil remained; and then the doctor, to get rid of this troublesome guest, took care that the shock should be strong enough to knock the patient down, and he was entirely cured.—*Armstrong's Lectures.*

### HYPOCHONDRIASIS IN MEDICAL PUPILS.

You will seldom be alarmed at hypochondriasis when it occurs in young subjects. I have, since I have lectured here, had the honour of curing some of the pupils of extraordinary dangerous organic diseases by very slight means. I have cured an aneurism of the aorta by a slight purgative; ossification of the heart by a little blue pill; and chronic disease of the brain by a little Epsom salts. You sometimes meet with such cases in private practice, and they are all cured by the removal of the bodily disorder or disease.—*Ibid.*

### PARALYSIS OF ONE SIDE OF THE FACE.

January 11th, 1824, I was aroused at an early hour in the morning by an application to visit a female in labour, who lived at the foot of Blackstone-Edge, a distance of four or five miles from Rochdale, my then place of residence. The case being one of emergency, and being obliged to dress in haste, I merely wrapped a thin cravat around my neck, neglecting the other usual coverings. On my journey I was exposed to a cold sharp wind, from which I felt much chilled about the face and neck. After being in the house an hour the labour terminated, when I remained to partake of tea with the female attendants, during which some anecdote was related that excited laughter, when, to my great surprise, I suddenly felt a peculiar sensation, and a numbness, in the left side of my face. I immediately turned to a mirror, and to my dismay perceived the whole of that side drawn completely awry, and presenting a most ludicrous appearance. Prior to this attack I had enjoyed uninterrupted good health; age twenty-five, person spare, and habits strictly abstemious. For nearly two years the left angle of the mouth had so completely lost its power, as to render it difficult during mastication to retain the food when on that side. I was also incapable of perfectly closing the eyelids of the affected side. I used a variety of remedies suggested to me by professional friends, without benefit, excepting what I derived from simple friction, which I daily employed with the flesh-brush, and which I have every reason to believe was advantageous. Ten years have now elapsed since the attack, and I am perfectly recovered, having suffered the paralysis about eight years, without the slightest symptom of a relapse.

Reflecting on this case, I should say, that the cause was not dependent either on disease of the brain or the digestive organs; but that it originated solely in an idiopathic affection of the portio dura, arising from exposure to cold.—*Mr. Rayner, in Lancet.*

### EMPYEMA CURED BY AN OPERATION.

BY J. PANCOAST, M.D.

William Smith, aged twenty-two, a shoemaker by occupation, took a severe cold in the winter of 1828-9, which was attended with severe cough, painful respiration, pain in the left side, and all the ordinary symptoms of pleurisy. He was relieved by his physician of the most urgent of these symptoms, but still retained, throughout that season, a continued uneasiness in his side, occasionally aggravated when he was more exposed to the inclemencies of the weather. At this period he continued at his work, as is usual with shoemakers, in a heated stove room, not complaining much, but feeling occasional faintness and shortness of breath, and walking (as his family observed) with his left shoulder so much depressed as to give him a lop-sided appearance. The succeeding summer he spent among his friends in the country, alternately better and worse, unable to attend to business, and apparently suffering with pulmonary consumption.

The winter following, 1829-30, all the symptoms were aggravated; he was confined to the house and subjected to medical treatment, but without any amelioration of his sufferings.

About June, in the following summer, the dyspnœa and pain of the side were much aggravated. He grew unable to sustain even slight fatigue, and was obliged, after ascending a flight of stairs, to repose for some time on a bed. He was so little inclined to exertion, that the utmost importunities of his friends could scarcely, during the whole summer, persuade him out of sight of his own door. The hectic symptoms with which he had for some time been affected, were now fully confirmed. He had several flushes of fever during the day, and was usually bathed in a cold sweat at night. The soreness and pain of his side became concentrated in a spot, the size of a man's hand, between the tenth and eleventh ribs. Upon this spot, about the middle of July, he received a bruise, which was a little painful at the time, but soon overlooked. Shortly after this, an external tumour was developed, the size of a common saucer; this was poulticed under the direction of a physician, and burst suddenly in the month of August, discharging according to the account of the family, about half a gallon of greenish fetid pus, with filaments of coagulated lymph. Exhaustion and syncope followed, and for a day or two he was in a state of excessive debility. The pain and oppression previously felt was however much relieved by the discharge.

I saw him for the first time in September; his side, which then presented three small openings, still continued to discharge drops of very fetid matter. The oppression and dyspnœa had returned to a worse degree than ever, and his appetite, which had previously been tolerably good, now failed him. He could only sleep when propt up in bed, and inclined upon the diseased side, and then but for a short space of time. Besides, hectic fever and sweats were fast dissipating the little strength he had left. He had a ticking sensation in his head, for which he had been directed by a physician a short time before to be bled, but which afforded no relief.

Upon examining the thorax, the ribs of the left side were found much sunken, measuring about an inch and a half less from the spine to the sternum, than those of the other side. The lower angle of the scapula was very prominent, the spine incurvated, the shoulder sunken, the heart palpitating in the right half of the thorax, where it seemed to have been forced by some fluid in the left. The whole of the left half of the thorax, except a small part below the clavicle in front, sounded dull, and with the stethoscope no respiration could be heard on that side, except near the clavicle. The right lung upon auscultation appeared healthy, but more resonant than usual, in consequence of the increased duty it had to perform from the useless condition of the other lung.

The symptoms of the case, connected with the fact of the previous discharge of purulent matter, left no doubt of a large accumulation of some similar fluid. The patient was nearly exhausted by the length and obstinacy of the affection, and therefore, to prolong his life even for a short time, it seemed necessary to resort to some means of removing the secretion.

Pus, when thus collected in the chest, unlike the serous effusions, is scarcely ever removed by absorption. The successive increase of the fluid reacts upon the membrane which forms it, and thus keeps up a perpetual irritation and a perpetual flow. If not withdrawn from the body, it compresses the lungs against the mediastinum, and destroys the patient by suffocation, or wears him out by the irritation.

Nature in some instances makes an effort for its discharge. An opening may be formed by ulceration through the pleura pulmonalis into one of the bronchia of the lung, and the matter be discharged by expectoration. In such cases, though relief for the moment is attained, the air in respiration makes its way into the cavity of the chest, becomes a new cause of irritation by its oxygen acting upon the pus, and in a short time the patient dies.

Another process of nature, and which is sometimes attended by success, is a tendency to point or form an opening externally through the parïetes of the chest. This process has been imitated by the hand of art, making an incision through the walls and discharging the fluid. But the air is apt here to pass in at the external wound and take the place of the secretion, and thus keep up an irritation, which, in the course of a month or two, usually destroys the patient.

Dupuytren has asserted, that out of more than fifty cases in which he had operated, he could quote but two instances of success; and Sir Astley Cooper, that he had never observed one.

Dupuytren has subsequently pursued a different plan, and which has proved much more successful. An analogous course has also been pursued by Baron Larrey. Its merit consists in an evacuation of the fluid without the introduction of air. This is done by the skin being first drawn upwards, and a trochar then pushed into the cavity, the fluid evacuated, and the skin allowed to take its natural position, and thus to close the opening. In this manner the operation is to be repeated at intervals of several days, as long as the fluid continued to accumulate.

In reflecting upon this case, it was evident that nature had done all that she could do towards the recovery of the patient, in giving the matter an external direction, and having caused a copious discharge. An operation now seemed required. It appeared to me, that even in the present emaciated and exhausted state of the patient, the chances of the patient's recovery was still probable, if the matter could be discharged without the introduction of air. The sinuses which had been left since the discharge in August, were found on probing to be very sinuous, and though they must have communicated with the internal abscess, a slow stillicidium of pus took place through them, and appeared to prevent the passage of air inwards. I therefore determined, in preference to Dupuytren's plan of repeated tapping, to imitate nature, by making a valvular incision into the cavity, and keeping it open by the frequent introduction of a catheter in an oblique direction. By this means I should be enabled to evacuate the fluid as it was secreted, and thus, by preventing distention again, allow the diaphragm a better chance to ascend, the lung to expand, and the ribs to fall inwards, so as to diminish the cavity of the abscess.

On the 27th of September, with the assistance of my friend, Dr. Condie, I made an incision in the middle of the space between the tenth and eleventh ribs, down to the pleura, and through this, which was covered on its inner surface with a thick coat of false membrane, a female catheter was pushed. About two quarts of pus with some odour was discharged in jets synchronous with the pulsations of the heart. The wound was then carefully closed, covered with a large piece of adhesive plaster and a compress, and the whole surrounded with a roller bandage. The patient was directed an infusion of bark and snakeroot, and the use of weak wine whey. The laborious breathing and the extreme general anxiety was immediately relieved. No faintness occurred from the discharges. In the course of a day or two the appetite was improved, the hectic symptoms much diminished, and natural slumber returned; at first daily, and subsequently at intervals of two and three days. I introduced a female catheter from the bottom of the wound obliquely upwards, to discharge the secretion, which usually amounted to from two to three gills. On the fourth day after the operation the heart had nearly resumed its natural position, and I discovered by the stethoscope that air penetrated into the lungs two or three inches below the clavicle. When the stethoscope was applied upon that side near the spine, the voice of the patient when he spoke appeared to come with great force through the tube, indicating that there was still within a large unoccupied cavity.

December 4th. Saw him again with Dr. Condie, every way improved; no night sweats; sleeps well; feels no pain; breathes easy. Introduced catheter again, and took away half a pint of bland serous pus. Heart regained its natural position. By the stethoscope, we discovered that air now passed down the left lung for four or five inches below the clavicle. When the stethoscope was placed close to the spine, any where opposite the thorax, a faint sound like the tintement metallique was occasionally heard. It was also heard at times two or three inches below the outer margin of the clavicle. This at one time gave rise to the opinion that there was a fistulous communication between the lungs and the cavity of the pleura. It was more probably owing to the plastic matter covering the pleura giving way during the partial expansion of the lung. Broncophony very strong below the clavicle; and during respiration there was occasionally heard a sound down the spine like the bleating of a goat, (ægophony.)

7th. The weather had suddenly changed, and the patient had been exposed to currents of air; countenance paler; spirits sunk; pulse small, palpitation of the heart heard more distinctly than before all over the left chest. Respiration much less distinct in left lung than at last visit. Introduced a gum elastic catheter, as the canal had become too winding for the silver, and took away half a pint of fluid, same colour and consistence as before. Directed the patient to be placed in a warm stove room, to be more warmly clad, to have his chest well rubbed with a strong liniment, and to take his tonic medicine more freely.

10th. Patient much improved; rests better; but has had considerable night sweats; respiration could now be heard in the left lung six inches below the clavicle, and down the whole length of the back; removed about half a pint of thin puruloid secretion. No pain on the introduction of the catheter. From this period he continued regularly to improve; the lung slowly expanding, and the quantity of secretion becoming less and less; care was taken to keep the orifice open, and a small catheter was introduced once a week, by twisting it through the winding canal into the cavity of the chest. The amount taken away was usually about a gill. Whenever a cold change of weather ensued, or the patient was more than usually exposed, the quantity was increased. The following June he was exposed to a shower of rain, and suffered from an attack of catarrh, to which affection he had for some time seemed very much disposed. While this lasted, the discharge consisted of thick bland pus, about three gills of which were evacuated every four days.

Counter-irritation was continually kept up upon the chest. After the tartar emetic eruption had disappeared, blister plasters were applied alternately to the back and front of the chest. Tonic remedies were continued. The patient walked and rode every day when the weather would permit.

July 1st. Examined him very attentively again with the stethoscope and pleximeter. Both indicated the presence of fluid at the bottom of the left pleural cavity. A mucous rhonchus was heard in the left chest like that of catarrh; and at the end of every inspiration, and sometimes in the middle, a sound like that of *ek*, pronounced in a strong inspiration. The left shoulder was considerably sunken; the spine was considerably curved with the convexity to the right side. The left side of the chest measured one and a half inch less than the right.

During the latter period of the treatment, when, from the development of the lung, rising of the diaphragm, and the reoccupation of its proper position by the heart, the superficies of the abscess was much diminished, I tried the measure advised in such cases by Dr. Cartwright, of Natches, to avoid the trouble and pain arising from the frequent partial introduction of the catheter, to prevent the sinus closing its whole length. This measure consists in the introduction of a bent wire into the cavity of the abscess, to act as a sort of syphon, by producing a discharge guttatim of the matter within. I found it difficult with it to exclude the air; it appeared also to irritate the lining membrane; and I was obliged to abandon it, and trust to the former mode, which had proved successful.

From this period the patient continued to improve. In the autumn, a year after he came under my care, he was able to resume his occupation. The sero-purulent secretion however still continued to form for near a year longer, and the patient's mother was in the habit every week or ten days, of introducing a catheter, and evacuating it to the amount of three or four ounces. As the amount decreased considerably, the canal for the first time was allowed to heal throughout its whole extent, for the first time, in August, 1831. At the present time the patient is in the enjoyment of as good health, and nearly as able to undergo fatigue, as at any previous portion of his life.

The gratifying results of the treatment of this case of empyema, a result so seldom met with when paracentesis thoracis is performed, appears due in part to the youth of the patient, and to the recuperative efforts which nature had already made, in effecting a discharge externally. But a great deal still appears to be owing to the valvular opening which was made, and kept open for so long a period, by which we were enabled to keep the secretions from accumulating in the chest, and thus allowing the walls of the immense abscess to approach each other, and finally to obliterate the cavity. In the numberless introductions of the catheter, a single bubble or two of air was the most ever allowed to enter, and then at the moment of its withdrawal. I note this case, in hopes that this process may be thought worthy of a trial by some more experienced hand.

A case of abscess of the chest, of some interest, occurred a few years ago in the upper part of this state, in the practice of a medical friend, and was cured in a way somewhat unique, which would seem to make it worthy of recital.

A man was seized with some affection within the thoracic cavity, which resisted the prescriptive treatment of his physician. The stethoscope at that period had not come into general use, and the case was so ambiguous, that its character was not revealed by its symptoms. The patient and his friends entertained the opinion that he was labouring under pulmonary consumption. No swelling, no discoloration, existed externally upon the thorax; but the patient, from the internal sensation produced by the disease, had a settled conviction that there was a gathering within. Taking a seat by an unfrequented side of the house, he plunged the blade of his penknife opposite the seat of pain between the ribs. He was found with pus flowing from the wound; a large quantity was discharged from the narrow wound. From that time his symptoms were relieved, and the patient finally recovered.—*American Journal of the Med. Sciences.*

### CHOREA SANCTI VITI.

Since my former communication in your journal on this disease, I have treated another case of it. A brother, aged seventeen, of the young lady whose case is there described, was attacked May 20th, 1832, in one side. His health was perfectly good, and no clue as to the seat of the latent irritation causing the disease could be ascertained. Without administering even a cathartic, or any other medicine, he was put on the use of the pulverised root of the Actea racemosa, a teaspoonful three times a day. He was getting worse from day to day before commencing the use of it, but it appeared to arrest its progress almost at once. After using it only two days he was visibly benefited, and was entirely cured in five days. He remains well, and no one of those formerly treated with it have had any return.—*Ibid.*

### LITHOTOMY IN ITALY.

It appears, from a table of the results of lithotomy at Naples, that the mortality from this operation in the hospital is 1 in 7; but that, in private practice, in the skilful hands of Petrunti, Santoro, &c., the deaths are only 1 in 20, or even 1 in 25. This is very different from the results in Paris, where, if M. Civiale is correct, 4 are lost out of 10. M. Dupuytren, wishing to know the reason of this extraordinary difference, was desirous of seeing the Italian surgeons operate. The clinical ward of surgery in the Naples hospital fortunately contained two persons suffering from calculus, and the director, Signor de Horatiis, operated upon them before M. Dupuytren, but not following precisely the Neapolitan method; in one case he employed Scarpa's lithotome, as modified by Signor de Horatiis himself, and in the other, Cheselden's knife.

The subject of the first operation was a countryman, aged forty, of a temperament compounded of the bilious and the sanguine; he had begun to suffer from the stone six years before, but the pains had increased extremely for the last year. He was cut in Cheselden's manner; the stone was of a moderate size. There was neither fever nor reaction, nor any symptom of cystitis. When the account came away, he was regularly progressing towards a cure.

The patient in the second case was a sawyer, aged nineteen, of a sanguine temperament, who had been afflicted with stone since his childhood, and had suffered severely for six months. He had had ischuria, and hematuria, and several small stones had passed by the urethra. He was cut with a modification of Scarpa's cutting gorget, and a stone of moderate size was extracted. He passed three lumbrici the same day, and in the evening there was considerable fever; some symptoms of cystitis afterwards appeared, which were kept down by baths. At the end of a week there was not a bad symptom, and his cure was proceeding quite regularly.

Cheselden's method is well known, but we will just say a word of the plan adopted in the second case. The external incision having been made just as in the lateral operation properly so called, the membranous portion of the urethra was exposed immediately above the bulb, for the space of four or five lines. The operator then holding the catheter perpendicularly in his left hand, and having the gorget in his right, introduced the beak of the latter into the groove of the former, pushed the gorget as far as the *cul-de-sac* of the groove, and then thrusting it forwards and upwards, penetrated the bladder. By this method the isthmus of the prostate is entirely divided above, and the veru montanum perpendicularly; a line and a half of the bladder being cut at the same time. The catheter being then withdrawn, the forceps are introduced along the concavity of the gorget, and the gorget is withdrawn with the right hand in the same direction.

These two operations were performed with the hand of a master, and M. Dupuytren loudly testified his satisfaction at the skill shown by the operator. It is clear that the plan adopted by M. de Horatiis is a modification of the method proposed by Thompson in 1808, and by M. Dupuytren himself in 1816. It has the great advantage of avoiding both the rectum and the pudic artery. Nevertheless there are inconveniences attending it, which have caused its disuse both in France and England.

But M. Dupuytren was particularly desirous of seeing the Neapolitan method, properly so called, put into practice by Santoro and Petrunti, in whose hands it is extremely successful. As there was no opportunity of operating on the living subject, Professor Santoro demonstrated this operation on the dead body. This was done publicly in the anatomical amphitheatre of the Cavalier Nanula, at San Francesco, in presence of M. Dupuytren, a number of Neapolitan professors, and a great concourse of pupils. The Neapolitan method consists in cutting obliquely (as Moreau did), and in giving the incision the form of two triangles, the base of the interior one being at the neck of the bladder and of the external one at the incision of the integuments; the apices of the two triangles meeting in the space which separates the perineum from the neck of the bladder. An oblique incision is therefore made from the raphe to the ischium, and by cutting down upon the catheter in the membranous part of the urethra, the external triangle is completed. The handle of the catheter is then to be depressed with the left hand, and the lithotome is plunged by the right hand obliquely upwards, and from left to right, following the grooves of the catheter; and when it reaches the bladder the neck of the bladder and the prostate are cut, completing the internal triangle.

The French professor having observed the simplicity of the operation with obvious marks of satisfaction, declared that at Paris the lateral operation was performed exactly in the same way as at Naples, and therefore that he had no objection to make, but that he had some questions to ask. 1st. How is it that, with the same method of operating, the proportion of fatal cases is so different at Paris and Naples? Professor Santoro answered, that the chief reasons are two in number; the difference of the climate, and the time of year chosen for the operation. The climate of Paris is much colder than that of Naples, and not only predisposes persons to inflammatory reaction, but develops it with great rapidity after operations attended with loss of blood. Moreover, from the coldness of the climate, inflammations of the bladder being more frequent, this organ becomes the seat of a chronic morbid diathesis, which is increased by the presence of the stone, and makes the development of inflammation after lithotomy more likely. As to the season chosen for the operation at Naples, excepting circumstances are urgent, lithotomy is performed only in spring and autumn, when, from the temperature, it is improbable that any atmospheric influence will affect the results of the operation; while at Paris surgeons operate at all times, even in the heart of the winter.

M. Dupuytren was satisfied with this answer, and said that he was of the same opinion, and was inclined to introduce this method of selecting the time of year at the Hôtel Dieu at Paris.

His second question was, What are the circumstances which most usually are the cause of death in patients at Naples? Professor Santoro answered: Death is never caused by hemorrhage, as every artery of any considerable calibre is avoided in the operation; but it generally arises from inflammation and suppuration of the kidneys, when they are already affected before the operation; sometimes, but rarely, from inflammation of the urinary passages, or the peritoneum; lastly, there have been years when those who have undergone lithotomy in the hospital have been attacked with gastric verminous fever, prevailing epidemically. As for myself, added the professor, I have never lost a patient when the operation has been performed as I wished: I have merely lost a few of those in whom, from some unforeseen accident, the steps of the operation were neither easy nor regular.

M. Dupuytren then observed, that the causes of death at Paris were the following ones, in the order of their frequency:

1st. Inflammation of the cellular tissue of the pelvis;

2dly. Inflammation of the urinary passages;

3dly. Peritonitis;

4thly. Gastro-enteritis.

As to the influence of the gastro-verminous epidemic, his opinion was, that gastric fevers were themselves a consequence of the operation; and that the presence of worms, which often occurs in similar cases, is merely a complication.

Professor Santoro replied, that, in the climate of Naples, gastric fevers are very common, and often put on an epidemic character. Thus, in the years 1827 and 1830, when this morbid complication caused the death of a number of patients operated on in the lithotomic ward, the whole town was full of these fever cases, and only a small minority of the population escaped the disease.

M. Dupuytren, then entering into details, remarked, that the inhabitants of Paris, having to bear a temperature which was often below the freezing point, became more accustomed to cold, and therefore suffered less from its effects; while the inhabitants of Naples, being subject to continual atmospherical vicissitudes, must suffer from their deleterious influence. Thus, for instance, the smallest depression of temperature being sufficient to affect them, and make them take to their cloaks and great coats, this excessive susceptibility would make them feel more intensely the effects of an operation.

M. de Renzi undertook to answer this. It was not necessary, he said, to recall to so eminent a surgeon the physiological and pathological theory, that cold predisposes persons to inflammatory diseases by keeping up a certain irritability of the vascular system; while hot climates, on the contrary, produce a nervous irritability. Moreover, in a hot climate, the cutaneous system, being in continual activity, is more excitable, and more disposed to be affected by heat and cold. These circumstances, therefore, make the inhabitants of hot climates more susceptible of the vicissitudes of the air, and particularly of cold; but the cold is never sufficiently intense or lasting to produce in the system a permanent predisposition to inflammation. For these reasons, in summer, after some operations, particularly the one for strangulated hernia, tonic and clonic convulsions are more common than inflammations.

Here the discussion stopped.

Whatever opinion we may have on the minor points brought forward by either side, one important fact remains, if experience sanctions it among ourselves, namely, that the selection of the time and season has an immense influence on the mortality following stone operations, and probably many others also.

M. Dupuytren is at present at Rome, but is to return to Paris at the end of this month (March). We shall therefore soon have the opportunity of seeing him put into practice the new ideas which have been suggested to him by his discussions with the surgeons of Italy.—*Gazette Médicale.*

### CASES OF DISEASED NERVES.

Charles Holiday, æt. forty, from Kirkheaton, was admitted by Mr. Wilks on the 20th September. He looked stout and healthy, and complained of nothing but loss of power and sensation in his left upper extremity. It was greatly enlarged from the shoulder point to the finger ends, and possessed its natural colour and temperature. The muscles felt somewhat hard; and over the back of the carpus there existed a ganglion the size of half an orange; and on the front of the shoulder joint a large tense bag, apparently of a similar nature. From the great enlargement, the head of the humerus could not be felt, although it was clearly not in its proper site. He could move the limb a very little backwards and forwards, but not raise it up in the least. At the same time, it admitted of being raised over his head, and moved in all directions by others. Indeed, the roughest handling gave him no pain; and the natural points of the thumb and two adjoining fingers were as if sliced off, having been often burnt and otherwise injured, without his being aware of it. This loss of feeling had existed seven or eight years; and he attributed it to his having fallen asleep in the sitting posture, with his arm stretched out before him, and resting on its inner side on the back of a chair. How long he remained in this position he could not tell; but, when he awoke, the arm was numb, and remained so ever after. He could always, however, raise it up and use it until six days before his admission, when it suddenly became powerless in the reaping-field, and the hook which he at the time was using fell from his grasp. The swelling likewise only commenced from that period.

The tumour in front of the joint was tapped with a fine trocar, and a gill of colourless fluid drawn off. The humerus could then be readily reduced, but the weight of the arm immediately dislocated it again. The Tinct. Iodini was freely applied to the shoulder and arm, with the effect of greatly reducing the size; and he wore a bandage, so as to retain the limb in its proper place. At the end of his two months, it was found that the arm kept reduced when the bandage was removed; but still he had no command over it. In this state he left the hospital. About the end of January I again met with him. The arm was out of its place, and hung dangling by his side. It could not be kept reduced without the bandage, of which he had tired. The shoulder and arm were nearly of their natural size, but the fingers and whole hand remained stiff and swollen.

That the cause assigned by Holiday for his complaint was the true one, I can readily believe. The nerve, injured by the pressure against the back of the chair, had never recovered its functions; hence the loss of feeling. The dislocation, I am of opinion, took place first in the reaping-field, when he lost all command over the limb; and the pressure of the head of the humerus, in its new situation upon the vessels in the axilla, will satisfactorily explain why the enlargement should commence at that period.

I remember attending a case of paralysis of the face, produced by a similar cause to that of Holiday's arm. It occurred in the spring of 1825, in a middle-aged gentleman, a patient of the late much-lamented Mr. M'Gown of Skelmanthorpe. He had fallen asleep with the right side of his face resting upon the wooden arm of a sofa. When he awoke, he felt much pain about the ear, and over the right half of the face; and, in a few days, the mouth and cheek were greatly drawn over to the left side. He presented no other symptom of disease, and I had much satisfaction in assuring him that there was no cerebral mischief, but that the palsy depended upon an affection of the portio dura in front of the ear. With repeated leeching and blistering, the face slowly regained its former symmetry; and the gentleman is now in good health.—*Dr. Turnbull's Report of the Huddersfield Infirmary, in Ed. Med. and Surg. Journ. for January* 1834.

### CASES OF DILATATION OF THE STOMACH.

Dilatation of the stomach, like that of the bladder or heart, may occur as a symptom of diseases of very different characters. In some of these the distention is produced mechanically, but in others its causes escape our research.

*On the different lesions which accompany, or apparently give rise to, dilatation of the stomach.* Its most common cause is schirrus of the pylorus, occasioning partial obliteration of its orifice. In these cases, where the food has been retained in large quantities, a portion will be vomited. Nevertheless, the stomach may be dilated either simply, without any other morbid appearance, or it may be accompanied by ramollissement and ulceration (proceeding in a few instances even to perforation,) of its parietes; and, in other cases, by hypertrophy of one or all its tunics. The dilatation in schirrus of the pylorus does not always arise from obliteration of the orifice, but will be found under other circumstances.

Case 1st. A man who had suffered from disorder of the digestive organs, attended by frequent vomiting, for eighteen years, at last died suddenly. The stomach was found uncommonly distended, reaching at least a hand's breadth below the umbilicus, and capable of containing twelve pints of fluid. The parietes were intimately adherent to the liver, being much attenuated at the upper and cardiac portion, but hypertrophied to a slight extent in the vicinity of the pylorus. (*Obs. Johannis Davidis Maucharti de Anatome ex Ventriculo vitio imprimis defuncti.*)

Here the adhesions of the liver, and the attenuation of the walls of the stomach, would of course favour the accumulation of food in its cavity. M. Mérat (*Journal Général de Med. Chir. Pharm.*) attributes this dilatation to the cessation of the action of the pylorus, which he compares to the action of the ascending portion of the cæcum; but M. Duplay would rather attribute it to the extension of the disease to the submucous structure of the parietes of the stomach obstructing their action, than any obstruction of the pylorus, which can act only as a sphincter. In corroboration of this opinion reference is made to a case reported by Andral, in which the muscular fibres were destroyed to the extent of four fingers' breadth from the pylorus. When this part is the seat of schirrous induration, its action will be proportionately impeded. The following case exemplifies another cause of dilatation of the stomach, while the pylorus remains permeable.

Case ii. A woman, æt. twenty-three, suffering from constant vomiting and obstinate constipation, gradually wasted, and died in about nine months from the onset of the symptoms. On a post-mortem examination, the greater circle of the stomach was found to reach to the pubis; its internal mucous coat about the fundus was much softened, and the parietes were altogether much thinner than natural, especially the muscular tunic.

Here the dilatation of the stomach can only be referred to the tenuity and want of power of the muscular fibre.

From the preceding facts we may observe, that dilatation of the stomach may arise either from morbid adhesions impeding the muscular contraction, or destruction of the muscular fibre at that part where its action is most required, or atrophy of the whole muscular tunic. We can easily perceive that these different lesions may totally prevent the action of the stomach, and consequently that the substances introduced into its cavity may occasion its morbid distention.

Allusion is next made to several cases recorded by Lieutaud and Blancard, of sudden death attended by great dilatation of the stomach; but, as the account of these cases is extremely imperfect, but little information can be obtained from their perusal. One case, however, reported in the *Historia Anatomica Morborum*, deserves to be related.

Case iii. A man, æt. sixty-five, was admitted into La Charité, in the end of March, 1752, complaining of a sensation of weight and distention at the epigastrium, and constant nausea, with inability to vomit. He was also labouring under anasarca and ascites. After death, the stomach was discovered to be greatly dilated, the pylorus being perfectly healthy, but the rest of the alimentary canal preternaturally contracted.

Case iv. A man, æt. fifty-four, came under the care of M. Rayer, with symptoms of hypertrophy of the heart, nausea, and occasional vomiting. He died in a few days afterwards, when the stomach was found to be greatly dilated, the pylorus and tunics remaining healthy. The only remarkable circumstance about the orifices of the stomach was, that they were both on the same level, instead of the cardiac extremity being considerably above the pyloric, which alteration in the position, and the consequent difficulty of the contents of the stomach passing readily towards the pylorus on a plane no longer inclined, M. Duplay thinks may partially account for its dilatation. In such cases, however, where little or no morbid appearance is found, the author is inclined to believe that there exists a true paralysis of the viscus. Such were the opinions of Lieutaud and Chaupier, the latter of whom compares this dilatation not unaptly to the relaxed state of the scrotum in certain subjects.—*Abridged from the Archives Générales.*

### CASE OF CONSTIPATION, SUCCESSFULLY TREATED BY THE INTRODUCTION OF AIR INTO THE BOWELS.

BY GEORGE J. JANEWAY, M.D.

July 7th, evening, I was called to see H. M., who was attacked last evening with a severe pain in her abdomen, which continued the greater part of the night, and was relieved by laudanum and the application of a sinapism. She has had occurrences of the pain through the day. Two or three days ago, in consequence of imprudent exposure during her menstrual period, her menses were suddenly stopped. On the 5th inst. I had prescribed for her a bleeding, for an affection of the heart, under which she is labouring. She also took a dose of Epsom salt.

She now complains of severe pain around the navel, which is relieved by pressure; pulse full, hard, and frequent, such as is met with in hypertrophy of the heart; tongue moist, a little furred in the centre; skin of natural temperature; her cellular tissue is infiltrated with serum; abdomen somewhat swollen. Her pain was soon relieved by the exhibition of laudanum and ess. of peppermint; flannel wrung out in hot brandy and Cayenne pepper. Directed castor oil, ℥j.

July 8th. Her pain returned soon after I left her, and has continued. Her bowels are more swollen; pain as before; no pain on pressure in any part of abdomen; tongue, pulse, and skin as before. V. S. ℥xvj.; fomentations of hops; calomel, grs. xij.; oil four hours after. Her bowels have not been opened since the 6th.

Six, P. M. Bowels not open; vomiting occasionally; abdomen more tympanitic; pains more severe; respiration hurried and somewhat oppressed. Soda water; repeat calomel; injections of senna tea.

10th. Since the preceding date all the symptoms have gone on increasing in severity, for which I directed, at different visits, leeches and cups to abdomen, cups to small of back, injections of warm and cold water, emollient injections, calomel and senna tea, fomentations to abdomen, &c. all without success. Bowels are still confined, greatly distended with gas; pulse pretty much as before; tongue furred in centre and moist; skin nearly natural; breath excessively fetid, can be smelt at the distance of several feet; belches considerably; hiccup occasionally; vomits a dark, fetid, oily fluid; respiration frequent and oppressed; face pinched.

At this time I determined to try the effects of the introduction of air into the bowels. Accordingly I attached one end of a bladder to the tube of a bellows, while a clyster-pipe attached to the other end was introduced into the rectum. The bellows were used a few minutes; during their use a fetid gas escaped from the rectum, in such quantity as to be smelt in different parts of the room. Immediately after the removal of the bellows, the patient passed by stool a pint of very fetid, dark fluid, together with a considerable quantity of gas. She felt somewhat relieved. A short time after, the bellows were reapplied with similar effects. A drop of croton oil was then given, and another at the expiration of two hours; the bellows were used twice afterwards.

Evening. Patient is much relieved; has had six passages by stool since the morning; she passed at the same time great quantities of gas. From this time she speedily recovered from her bowel affections. Her stools did not become of a natural appearance till some days after.—*American Journal of the Med. Sciences.*

### TREATMENT OF DROPSY.

You should in all cases investigate the cause, whether it be inflammation, plethora, obstruction to the return of blood, or some change in the blood, or whether the dropsy be encysted. It is in vain to prescribe for a symptom without reference to a cause. Books tell us that the treatment of dropsy is very simple. They tell us that there are two indications; one is to evacuate the fluid, and the other is to prevent the recurrence of it. All this is very true, but dropsy, as far as its cause and its treatment are concerned, is very different. Some forms of dropsy are remediable; those arising from inflammation generally are so.

1. If dropsy be connected with inflammation, the urine is scanty and high-coloured, and on boiling it, or adding to it nitric acid, it very often, but not always, deposits albumen. Other general symptoms will guide you, as hardness and frequency of the pulse, a furred tongue, and a hot skin towards night. Bleeding, purging, and a regulated diet, are to be adopted in these cases. In some strong subjects you may bleed largely, but in delicate patients you must abstract a moderate quantity of blood. Other medicines greatly assist you in these cases, as digitalis, squill, or calomel. Digitalis may be given in infusion, two drachms morning and evening, gradually increased to half an ounce, six drachms, or an ounce, twice a day. Of the tincture of digitalis ten drops may be given twice a day, gradually increasing the dose. In giving digitalis attend to the state of the pulse, of the stomach, and of the head. If there be retching, or giddiness, or if the pulse become small and slower, omit the digitalis. If it produce very alarming effects, give ammonia and wine, or opium and brandy. Squill, when recent, operates remarkably well. It often fails as a diuretic, because it is not good. Colchicum is another remedy which has very great efficacy. I generally give colchicum twice or three times a day, with a purgative; it increases the flow of urine in inflammatory diseases very remarkably, and tends powerfully to carry off the inflammatory symptoms. When it occasions any degree of sickness, withdraw it immediately.

2. When obstruction to the return of the blood exists as the cause of dropsy, you must ascertain what is the condition upon which it depends, in order to know whether that condition is remediable. If it exist about the liver or bronchial linings, you may frequently relieve it: in the one case, by alteratives every second night, and daily purging by calomel, elaterium, or turpentine, with alkalies and a regulated diet; in the other, by purging, diaphoretics, and a regulated temperature. If the heart be obstructed, you must also attend to the liver: alteratives and purging, and moderate bloodletting, with a bland diet and rest, will be of great benefit. Sydenham was in the habit of giving emetics and nauseants in dropsy of the belly; they are seldom used now: but as ascites is often connected with torpor of the liver, when it arises from that cause, they may occasionally be employed with great advantage.

3. If the dropsy arise from repletion, and if that depend on a very large quantity of blood, you may bleed your patient, open his bowels, put him upon a moderate diet, and let him use a warm bath; and the dropsy disappears. Nauseants are sometimes productive of great advantage. If the repletion arise from the sudden absorption of a large quantity of water, use purgatives, the warm bath, and occasional alteratives, with rest, a spare diet, fresh air, and a regulated temperature.

4. Where it arises from some change in the blood, with laxity of the solids, you must endeavour to ascertain whether the affection from which that change is derived be a disorder or a disease. If it be a disorder of the stomach, liver, or bowels, as it often is, and you remove it by mild laxatives daily, mild alteratives every other night, fresh air, a bland diet, and bleeding or leeching, if requisite, the dropsy soon disappears. If there be organic disease, all that you can do generally is to palliate the disease. One palliative sometimes is the operation of tapping. Diuretics are sometimes useful.—*Armstrong's Lectures.*

### ON THE CROWING INSPIRATION OF CHILDREN.

This important disease, which was described by Dr. John Clarke, under the name of "a peculiar species of convulsion in infant children," has lately been the subject of several papers by Dr. Hugh Ley, in the Medical Gazette; and, as these essays are evidently the fruit of sound and repeated observation, we think it right to present our readers with a few of the more prominent points contained in them.

Dr. Clarke thought that this convulsion, accompanied by an interruption of the respiration, necessarily depended on cerebral derangement, and the practice adopted in consequence by himself and his followers, has been in many cases far too violent.

Underwood, Capuron, North, and others, however, have combated the truth of this doctrine, and have shown that, although this disease may in some cases depend on cerebral turgescence of irritation, yet that it may also frequently exist without them.

This sonorous inspiration may depend on disease of the thoracic absorbent glands; a fact first pointed out by Dr. Merriman, who says, "in two cases of this kind, which were under my care nearly at the same time, the children died in the fits. They were both opened by Mr. Sweatman, a very skilful anatomist, but not the slightest appearance of cerebral affection could be discovered in either of them. The principal deranged structure discovered was a collection of small glandular swellings in the neck, pressing upon the par vagum." (*Note upon Underwood*, p. 140.) Dr. Hugh Ley, however, who is in possession of the preparations taken from these cases, affirms that it was the recurrent nerves which were chiefly injured, and that in one of them the left recurrent, as it turns round the arch of the aorta, is raised more than half an inch; moreover, it is flattened by this elongation, and is altered in texture, appearing withered. Dr. Hugh Ley believes that this disease of the absorbent glands, and this crowing inspiration, are connected together as cause and effect, and narrates the following cases in proof. In the first, the child, who had been supposed by a very eminent physician to be labouring under hydrocephalus, grew worse under a great variety of treatment, and at last died. Mr. Arnott opened the body: the cluster of glands at the root of the lungs, and about the arch of the aorta, were greatly diseased; one of them, as large as a middle-sized chesnut, being full of the matter which is produced by scrofulous inflammation, and another smaller one having imperfectly suppurated. With the exception of a slight enlargement of some of the mesenteric glands, there was no other disease in any part of the body. In the second case there were vomicæ in the lungs, besides the enlarged glands, but the crowing inspiration had most probably depended on the latter.

Dr. Hugh Ley has also observed, that when these nerves are morbidly pressed upon, in their course upwards to their ultimate destination, namely, the muscles which move the arytænoid cartilages, a similar dyspnœa is produced. His final conclusion is, that the disease does not depend upon a spasm of the muscles which close the glottis, but rather on a paralysis of the muscles which open it.

Dr. Ley's treatment consists chiefly in tonics and gentle aperients; for quieting the cough he employs conium, or the hop, but prefers the latter; he also recommends the external application of an opiated liniment. He agrees with Mr. North in commending the frequent and effectual lancing of the gums, when these cases appear to depend on irregular dentition.

### THERAPEUTIC EFFECTS OF CODEINE.

We learn from the Gazette Médicale, that Dr. Barbier has tried the medicinal effects of codeine, a new alkaloid derived from opium.

The dose is one or two grains, and he administers it in syrup. He is of opinion, that, although codeine, like all the preparations of opium, acts upon the nervous system, yet that it has but little effect upon the brain, and none at all upon the spinal marrow, but that all its energy is displayed upon the nervous plexuses of the great sympathetic. It is in the epigastric region that its power is manifested; it is in this centre of the ganglionic nerves, says Dr. Barbier, that we can best appreciate its value. It is given with advantage to patients suffering from pain extending from the epigastrium to the back, accompanied with a sensation of burning, uneasiness, depressed spirits, &c. Whether this disease be called gastralgia, or pain in the stomach, or chronic gastritis, or abdominal neurosis, its situation is certainly in the nervous plexuses, and depends upon a morbid state of them. Dr. Barbier has seen the syrup of codeine cure these pains, as well as the accompanying symptoms; the gaiety of the patients, and the ease with which they turned in their beds, forming a strong contrast with the state of oppression and anxiety to which they had been subjected for months, indeed, in the case of one female patient, for a year. Dr. Barbier had several patients under his care at the Hôtel Dieu at Amiens, who, besides the abdominal neurosis just mentioned, were afflicted with neuralgic pains in the head, loins, or thighs: in such cases the codeine relieved the pains and uneasiness in the epigastric region, but had no effect upon other parts. The patients who derived benefit from the use of codeine had generally taken the liquid laudanum of Sydenham previously, without advantage.

Codeine does not produce any apparent change in the circulation, or breathing; it does not disturb digestion, but it rather diminishes the appetite: it often causes an itching of the skin; finally, it does not cause constipation. Mr. Wm. Gregory's experiments on codeine do not coincide with these; but he employed the nitrate, and, according to M. Kunkel, codeine loses much of its efficacy when combined with acids. The discovery of this alkaloid is due to the researches of M. Robiquet.

---

